# Collateral grade of the Willis' circle predicts outcomes of acute intracranial internal carotid artery occlusion before thrombectomy

**DOI:** 10.1002/brb3.1452

**Published:** 2019-11-07

**Authors:** Hongchen Zhao, Baolin Wang, Ganggang Xu, Yi Dong, Qiang Dong, Wenjie Cao

**Affiliations:** ^1^ Department of Neurology Huashan Hospital Fudan University Shanghai China; ^2^ The Third Peoples' Hospital of Qingdao Qingdao China; ^3^ University of Miami Coral Gables FL USA

**Keywords:** acute ischemic stroke, intracranial internal carotid artery occlusion, mechanical thrombectomy, primary collaterals, Willis' circle

## Abstract

**Background and Purpose:**

Endovascular mechanical thrombectomy (EVMT) shows significant promise in improving acute ischemic stroke (AIS) with proximal artery occlusion, but outcomes have been variable. We explored the patients treated by thrombectomy to investigate the association between a favorable clinical outcome of EVMT in intracranial internal carotid artery occlusion (iICAO) and a set of predictors.

**Methods:**

A total of 38 iICAO patients treated by EVMTs were analyzed. Primary collateral grades (PCG) at baseline based on the integrity of Willis' circle were categorized into three degrees. The favorable outcomes, measured by modified Rankin scale (mRS), were defined as ≤2 at 90 days. The reperfusion was one of the most important confounders, defined as modified thrombolysis in cerebral infarction (mTICI) ≥ 2b. The other risk factors included demographic characteristics, vascular risk factors, stroke severity, procedural EVMT, and PCG at baseline was adjusted to reveal the association with favorable outcomes.

**Results:**

Of 38 iICAO patients, 65.8% (25 in 38) achieved reperfusion. However, only 31.6% (12/38) achieved favorable outcomes at 90 days. With a PCG3, 61.5% of them achieved favorable outcomes, while only 37.5% of those with PCG2 and PCG1 achieved favorable outcomes (*p* = .003). In multivariable logistic regression, PCG was revealed as a predictor for favorable outcomes (OR 5.278, *p* = .019) after adjusting the reperfusion and other factors.

**Conclusions:**

The PCG based on the integrity of Willis' circle might be an underlying predictor of the prognosis of AIS in patients with iICAO after EVMT. The function of intact anterior communicating artery (AcoA) and ipsilateral posterior communicating artery (PcoA) in favoring prognosis of the iICAO patients might need to be validation in future study.

## Graphical abstract

PCG, when catalogued according to the integrity of the circle of Willis, had a strong power to predict prognoses in AIS patients with iICAO treated by EVMT, and favorable functional outcomes were more often achieved in patients in whom both the AcoA and ipsilateral PcoA were preserved.

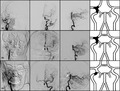

## 1 INTRODUCTION

Endovascular mechanical thrombectomy (EVMT) is proven benefit in patients with acute ischemic stroke (AIS) with anterior circulation large vessel occlusion. A rate of favorable outcomes of 32.6%–60% was achieved in the intervention group of the MR CLEAN trial (Berkhemer et al., 2015) and the SWIFT PRIME trial (Saver et al., 2015). However, different the rate of favorable outcomes varying from 28% to 60% (Fockaert et al., 2015; Hong et al., 2016; Kim, Kang, Hwang, Park, & Kim, 2016; Kwak et al., 2014) were reported, suggesting that a substantial proportion had not recovered independence by 90 days after onset. Several factors, including National Institutes of Health Stroke scale (NIHSS) onset, occlusion type and occlusion level, have been reported to be associated with favorable functional outcomes, while others, such as poor initial status, unsuccessful recanalization, cardioembolism, and supraclinoid‐terminal occlusion, are associated with poor outcomes of AIS with internal carotid artery occlusion (Kwak et al., 2014). Furthermore, collateral is another important factor that may potentially impact prognosis. In the SWIFT PRIME trial (Jadhav et al., 2017), MR CLEAN trial (Berkhemer et al., 2016) and ESCAPE trial (Goyal et al., 2015), pial collateral status according to CTA, were found to positively relate with favorable functional outcomes of AIS with anterior circulation LVO. Additionally, it has been demonstrated that the presence of bilateral posterior communicating arteries (PcoAs) was a predictor of a favorable functional outcome in the patients with basilar artery occlusion (Maus et al., 2017). As was expected, Willis' circle plays an important role as the primary collateral in nature, might be essential in patients with ICA occlusion. However, little was known about the association between the integrity of Willis' circle and favorable outcomes in patients with ICA occlusion.

Therefore, we established a strategy to evaluate the integrity of Willis' circle by categorizing it into primary collateral grades (PCG) in AIS patients with iICAO and investigated the association between PCG and prognosis in patients treated with EVMT.

## 2 METHODS

A retrospective analysis of consecutive patients with acute ischemic stroke treated by EVMT was performed on data entered into a prospectively captured database at a senior stroke unit from April 2015 to February 2018.

All patients suffering from intracranial internal carotid artery occlusion were included. Patients eligible for treatment with intravenous (IV) recombined tissue plasminogen activator (rt‐PA) within 4.5 hr of stroke onset were treated with intravenous thrombolysis first and then bridged to interventional therapy; other patients were treated with EVMT directly. The anesthesia model was determined by operators according to the state of the patient's consciousness and airway: in patients who were unconsciousness and at risk of airway obstruction, general anesthesia was maintained during the procedure, while others underwent the procedure under conscious sedation. Contralateral carotid artery angiography and priority vertebral artery angiography were manipulated to identify the collateral circulation, including the integrity of Willis' circle and the pial collateral. Stent‐retrievers (Solitaire AB, Covidien/ev3) were deployed and retrieved as the device routinely used for EVMT. The maximum number of attempts and indications of dual‐stents retrieval was up to the discretion of the operators.

**Figure 1:**
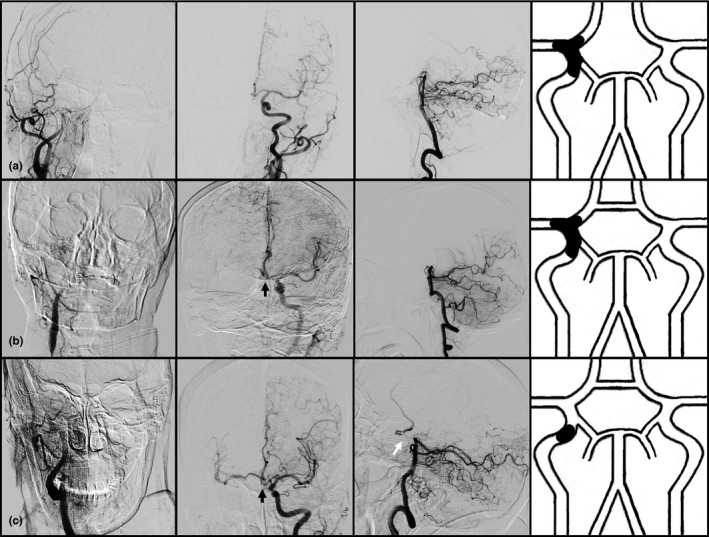
Illustration of primary collateral grades (PCGs). Row a: In PCG 1, the anterior communicating artery (AcoA) was absent regardless of whether the ipsilateral posterior communicating artery (iPcoA) was preserved (illustrated as dotted line); Row b: In PCG 2, the AcoA in preserved, but the iPcoA is absent. Row c: In PCG 3, both the AcoA and iPcoA were preserved. Black arrow ‐AcoA, White arrow ‐iPcoA

According to the integrity of Willis' circle, as presented on digital subtract angiography (DSA), PCG was categorized as 1 of 3 classes (Figure 1): in grade 1, the anterior communicating artery (AcoA) was absent regardless of whether the ipsilateral posterior communicating artery (iPcoA) was preserved; in grade 2, the AcoA was preserved but the iPcoA was absent; and in Grade 3, both the AcoA and iPcoA were preserved. A preserved AcoA was defined as the observation of collateral flow from the contralateral internal carotid artery to the ipsilateral anterior circulation (either the anterior cerebral artery region or the MCA) as verified on angiography of the contralateral carotid artery, and a preserved iPcoA was defined as collateral flow from the posterior circulation to the ipsilateral anterior circulation as verified on angiography of either vertebral artery.

Data, including demographics, cardiovascular risk factors, cerebrovascular events, comorbidities, severity of stroke (NIHSS), time of onset to groin puncture, reperfusion degree (modified thrombolysis in cerebral infarction, mTICI), and clinical outcome (modified Rankin Scale, mRS) at 90 days after onset, were collected. The NIHSS parameters were assessed by stroke neurologists with the certification issued by the American Heart Association (AHA). The mRS was assessed by neurologists using a structured interview to obtain mRS scores in the outpatient department for patients who were mobile or by telephone interview for patients who were bedridden. The PCG and mTICI were assessed by a neurointerventionist and a radiologist according to the DSA imaging, and a senior consultant was involved to evaluate the scales described above if they did not reach an agreement.

All statistical analyses were conducted with SPSS software (version 20.0). Univariate analysis was performed using Fisher's exact and *χ* ^2^ test for categorical variables and the two‐sample *t* test or Mann–Whitney *U* test for continuous variables to determine the distribution patterns of baseline factors, interventional parameters, and outcomes. All variables with *p* < .10 in the univariate analysis among groups of different clinical outcomes were included in subsequent analyses performed using multivariable logistic regression to investigate independent predictors for favorable functional outcomes and mortality. *p* < .05 was defined as statistically significant. Akaike information criterion (AIC) and Bayesian information criterion (BIC) were calculated to estimate the relative quality of statistical models.

This study was a retrospective study, and data collection was approved by the Institutional Review Board of Huashan Hospital.

## 3 RESULTS

### 3.1 Baseline characteristics

During the period from April 2015 to February 2018, a total of 105 patients received endovascular treatment for AIS with LVO. Of these patients, 38 (36.2%) with culprit iICAO were included in this case–control study. Patients suffered iICAO presented severe symptom onset, with a median of NIHSS 23, and 42.1% (16/38) of them received IV rt‐PA due to the delay from onset to arrival at hospital.

### 3.2 Primary collateral grades

Based on DSA, AcoA was absent in 17 (44.7%) of the 38 patients, and these patients were subsequently cataloged as having grade 1 primary collateral circulation in iICAO. Among the remaining patients with a preserved AcoA, 8 (21%) had no iPcoA and were categorized as having grade 2 primary collateral circulation, while the remaining 13 (34.2%), who had both an AcoA and an iPcoA, were categorized as grade 3 for primary collateral circulation.

### 3.3 Predictive factors of outcomes

Univariate analysis showed that patients with unfavorable outcomes were significantly older than those who achieved independence at 90 days after onset (79.5 vs. 68.5‐years old, *p* = .015). Furthermore, patients with unfavorable outcomes had more severe symptom onset, with a median NIHSS of 25.5, than that in 18 independent patients (*p* = .001). Finally, successful recanalization, which was defined as mTICI ≥ 2b, occurred more frequently in the favorable outcomes group, and the proportion of patients categorized as grade 3 patients was also higher in the favorable outcomes group (*p* = .005) (Table 1).

**Table 1:** Univariate analysis among groups with different clinical outcomes

Clinical outcome	Total （*n* = 38）	Favorable (*n* = 12)	Unfavorable (*n* = 26)	*p* a
Gender (M), no. (%)	21 (53.30)	8 (66.67)	13 (50.00)	.337
Age (years), median (IQR)	77.5 (69.0–81.0)	68.5 (50.5–76.75)	79.5 (74–82)	.015
Hypertension, no. (%)	25 (65.80)	6 (50.00)	19 (73.08)	.163
Diabetes, no. (%)	5 (13.20)	0 (0.00)	5 (19.23)	.103
Atrial fibrillation, no. (%)	22 (57.90)	8 (66.67)	14 (53.85)	.457
NIHSS (preop), median (IQR)	23.0 (18.0–29.25)	18 (15–22.50)	25.5 (21.75–31.75)	.001
ASPECTS, median (IQR)	7.5 (6.0–9.0)	8 (6–9)	7 (6–8.25)	.466
Blood glucose (onset), mean ± *SD*	7.97 ± 2.27	7.28 ± 2.01	8.29 ± 2.34	.206
SBP (onset), mean ± *SD*	154.37 ± 29.53	148.83 ± 22.79	156.92 ± 32.25	.440
DBP (onset), mean ± *SD*	83.45 ± 16.26	83.00 ± 18.62	83.65 ± 15.45	.910
IV rt‐PA, no. (%)	16 (42.10)	6 (50.00)	10 (38.46)	.503
Onset puncture time, median (IQR)	345 (236.75–391)	326 (213–451)	349 (245–388.50)	.816
Primary collateral grade, no. (%)				
1	17 (44.70)	1 (8.33)	16 (61.54)	.005
2	8 (21.10)	3 (25.00)	5 (19.23)	
3	13 (34.20)	8 (66.67)	5 (19.23)	
mTICI ≥ 2b, no. (%)	25 (65.80)	11 (91.67)	14 (53.85)	.022

a Comparison between patients with favorable and unfavorable outcomes.

After adjusting for gender, comorbidities, Alberta Stroke Program Early CT Score (ASPECTS), recanalization, and time interval from onset to recanalization, multivariable logistic regression analysis revealed that PCG was positively associated with favorable neurologic functional outcomes at a significance level of 0.10 but not 0.05 (Table 2, model 1).

**Table 2:** Multivariable logistic regression analysis of the association between PCG and favorable outcomes (mRS ≤ 2 at 90 days), adjusted or not for NIHISS scores

	mRS (90 days) ≤2, OR (95% CI)	*p*	AIC	BIC
Model 1				
Age	0.887 (0.772–1.020)	.092	34.628	35.648
Preoperative NIHSS	0.850 (0.693–1.044)	.121		
Recanalization (mTICI ≥ 2b)	6.052 (0.348–105.183)	.217		
Primary collateral grade	3.331 (0.781–14.204)	.081		
Model 2				
Age	0.887 (0.780–1.008)	.065	34.997	35.762
Recanalization (mTICI ≥ 2b)	8.385 (0.623–112.937)	.109		
Primary collateral grade	5.278 (1.318–21.131)	.019		

Note

In model 1 of the multivariable logistic regression analysis, the primary collateral grade was analyzed along with age, recanalization, and preoperative NIHSS.

In model 2 of the multivariable logistic regression analysis, the primary collateral grade was analyzed along with age and recanalization but not preoperative NIHSS.

In view of the significant association between NIHSS scores and PCG (Kendall's tau = −0.37, *p* = .001), NIHSS score might have a similar impact on predictions of the prognosis of intracranial carotid artery occlusion treated with endovascular thrombectomy. Logistic regression model (Table 2, model 2) in which NIHSS scores were removed confirmed that PCG was an independent predictor for outcomes (OR 5.278, *p* = .019). Additionally, Akaike information criterion (AIC) and Bayesian information criterion (BIC) of the two models promised similar quality (Table 2).

## 4 DISCUSSION

Collateral status, beyond age, diabetes, NIHSS onset, ASPECTS, and recanalization, can be used to determine infarction and ischemic penumbra volumes (Rusanen, Saarinen, & Sillanpaa, 2015) and was demonstrated to be positively associated with favorable functional outcomes in both the MR CLEAN trial (Berkhemer et al., 2016) and the ESCAPE trial (Goyal et al., 2015). Furthermore, after correcting for stroke severity, Agarwal found that good collateral status was the only independent predictor of improvement in clinical status (Agarwal, Bivard, Warburton, Parsons, & Levi, 2018). However, we noted that pial collateral status is usually evaluated as a predictor of outcomes in AIS patients. Few studies have focused on the impact of Willis' circle on clinical outcomes, even though a complete Willis' circle was found to indicate not only better functional outcomes but also less hemorrhage transformation (Chuang et al., 2013; Lee et al., 2016). Among AIS patients with LVO, unfavorable anatomical variations in Willis' circle, such as a hypoplasticity or aplasticity of the contralateral A1 segment and ipsilateral P1 segment, could predict poor clinical outcomes (Bradac et al., 2017). Moreover, in the case of iICAO, pial collaterals could be affected by the integrity of Willis' circle, and it might therefore be more direct to evaluate collateral status by grading the integrity of Willis' circle.

We established an accessible strategy to grade the integrity of Willis' circle by DSA during the EVMT procedure and demonstrated that there is an association between functional outcomes and this grading scale. Our findings indicate that the presence of both the AcoA and the iPcoA predicted the best outcome among these three groups and that only the preservation of the AcoA predicted significantly favorable outcomes. A similar finding was presented in Silvestrini et al. (2011), in which the preservation of at least two activated intracranial collateral vessels (the ophthalmic artery, AcoA or PcoA) produced a significant trend toward favorable outcomes in patients with internal carotid artery occlusion due to dissection.

In contrast, in Sundaram's study, among patients suffering intracranial carotid artery occlusion, the odds of a favorable outcome at 90 days after onset was higher in the group with ipsilateral ophthalmic artery or leptomeningeal collaterals than in those with a complete Willis' circle (Sundaram, Kannoth, Thomas, Sarma, & Sylaja, 2017). However, in their study, they included patients who were up to 3 weeks after onset, and their patients differed from candidates for EVMT. Secondary collaterals (the ipsilateral ophthalmic artery or leptomeningeal collaterals) can vary dynamically over time and have a substantial impact on functional outcomes, while communicating arteries are intrinsic/underlying bypass vessels that establish immediate compensatory blood flow for the distribution of arteries occluded proximal to Willis circle and determine the volume of the penumbra more directly during the hyperacute phase of stroke. It was similarly demonstrated in this study that variation in Willis' circle modified the prognosis in AIS patients with iICAO.

Several methods, including CTA (Sundaram et al., 2017; Yeo et al., 2015) and MR (Alves et al., 2018), have been established to evaluate collateral status. Although CTA is highly accurate in the assessment of anatomical variations of Willis' circle, its sensitivity remains limited in its ability to reveal hypoplastic segments (Han et al., 2011), and time‐of‐flight (TOF) MRA has disadvantages in a setting involving slow flow at the site of occlusion, which often results in complete loss of the signal in arteries and the consequential underestimation of collateral status (Raymond & Schaefer, 2017). DSA remains the most direct and most accurate technique for detecting the completion of the Willis' circle and is always regarded as a standard reference. When proceeding to EVMT, the deficit of invasion and risk of vessel injury associated with DSA are no longer disadvantages.

Both this study and that conducted by Zhou et al. (2016) revealed that the integrity of Willis' circle is strongly associated with stroke severity, hinting that having a complete Willis' circle exerts a protective effect in AIS. Actually, the circle of Willis plays an important role as the primary collateral interhemispheric and between anterior and posterior circulation. In ICA occlusion patients, an intact Willis' circle promises compensatory flow of blood from the contralateral hemisphere via the anterior communicating artery and from the posterior circulation via the ipsilateral posterior communicating artery. This compensatory blood supply could decrease the size of the infarction core, subsequently leading to a favorable prognosis. When the anterior communicating artery and/or posterior communicating artery is absent, insufficient compensatory pial collaterals from the posterior cerebral artery become the major blood support for the ischemic brain tissue supplied by the occluded carotid artery. This could result in more severe manifestations and worse outcomes.

Although the association between favorable functional outcomes and primary collateral grade was not significant after adjusting for NIHSS scores. However, Kendall's tau (−0.37, *p* = .001) indicated there was a mild‐to‐moderate correlation between NIHSS scores and PCG. Nevertheless, since the ternary‐graded PCG scoring system is more specific and practical than the severity scale of stroke, we established a logistic regression model in which PCG substituted NIHSS scores. Subsequently, the Akaike information criterion (AIC) and Bayesian information criterion (BIC) of the two models confirmed the validation and fitness of the predictive model. As an underlying determinant of stroke severity, the PCG was found to be significantly related to favorable functional outcomes in the multivariate analysis.

### 4.1 Limitations

Our study is limited by the retrospective nature of analysis and small number of patients from a single center, the further work of us is to validate our predictive model in large registry database. The retrospective design of this study may have been to selective bias as we excluded patients who refused endovascular thrombectomy. Furthermore, stroke etiology was not separated for analysis in this study.

## 5 CONCLUSIONS

Primary collateral grades, categorized based on the integrity of Willis' circle, had a strong power to predict prognoses in AIS patients with iICAO treated by EVMT, and favorable functional outcomes were more likely achieved in patients in whom both the AcoA and ipsilateral PcoA were preserved.

## CONFLICT OF INTEREST

None declared.

## Data Availability

The data that support the findings of this study are available on request from the corresponding author. The data are not publicly available due to privacy or ethical restrictions.
